# Estimation of Dietary Intake of Radionuclides and Effectiveness of Regulation after the Fukushima Accident and in Virtual Nuclear Power Plant Accident Scenarios

**DOI:** 10.3390/ijerph15081589

**Published:** 2018-07-26

**Authors:** Michio Murakami, Takao Nirasawa, Takao Yoshikane, Keisuke Sueki, Kimikazu Sasa, Kei Yoshimura

**Affiliations:** 1 Department of Health Risk Communication, Fukushima Medical University School of Medicine, 1 Hikarigaoka, Fukushima 960-1295, Japan; takanira@fmu.ac.jp; 2 Institute of Industrial Science, University of Tokyo, 5-1-5 Kashiwanoha Kashiwa, Chiba 277-8574, Japan; takao-y@iis.u-tokyo.ac.jp (T.Y.); kei@iis.u-tokyo.ac.jp (K.Y.); 3 Faculty of Pure and Applied Sciences, University of Tsukuba, 1-1-1 Tennodai, Tsukuba, Ibaraki 305-8577, Japan; ksueki@ied.tsukuba.ac.jp (Ke.S.); ksasa@tac.tsukuba.ac.jp (Ki.S.)

**Keywords:** cost-effectiveness analysis, Fukushima Daiichi Nuclear Power Station accident, food, internal dose, radiation risk assessment, regulation

## Abstract

Evaluation of radiation exposure from diet is necessary under the assumption of a virtual accident as a part of emergency preparedness. Here, we developed a model with complete consideration of the regional food trade using deposition data simulated by a transport model, and estimated the dietary intake of radionuclides and the effectiveness of regulation (e.g., restrictions on the distribution of foods) after the Fukushima accident and in virtual accident scenarios. We also evaluated the dilution factors (i.e., ratios of contaminated foods to consumed foods) and cost-effectiveness of regulation as basic information for setting regulatory values. The doses estimated under actual emission conditions were generally consistent with those observed in food-duplicate and market-basket surveys within a factor of three. Regulation of restricted food distribution resulted in reductions in the doses of 54–65% in the nearest large city to the nuclear power plant. The dilution factors under actual emission conditions were 4.4% for radioiodine and 2.7% for radiocesium, which are ~20 times lower than those used in the Japanese provisional regulation values after the Fukushima accident. Strict regulation worsened the cost-effectiveness for both radionuclides. This study highlights the significance and utility of the developed model for a risk analysis of emergency preparedness and regulation.

This paper was prepared after an English translation and the addition of re-analyses, results, and discussion to abstracts presented at annual meeting/conferences [1,2,3].

## 1. Introduction

Internal exposure from diets is one of the pathways of radionuclides at nuclear power plant accidents [4,5]. The thyroid doses among evacuees after the 1986 Chernobyl accident (490 mGy in the first year) were mainly derived from diets and contributed to the increase in thyroid cancer occurrence in young children [4]. On the contrary, there were limited doses from both internal and external exposures in the 2011 Fukushima accident [5]. In particular, the internal exposure from diets was very minor (e.g., the first-year exposure in Fukushima City: 0.84 mSv of the thyroid equivalent dose) [6], partly because rapid measures such as regulations on food distributions, which were previously prepared in accordance with the lessons learned from the Chernobyl accident, were implemented just after the accident. In contrast, strict regulation has caused economic damage to specific industries [7]. It is important to assess the exposure from diets and the effectiveness of regulatory measures toward the establishment of regulatory values under the assumption of a virtual accident as a part of emergency preparedness [8].

Numerical simulation models for the transport of a radioactive plume and deposition have been developed and applied to estimate the exposure and to establish evacuation plans for emergency preparedness [9,10,11,12]. To the best of our knowledge, however, integrated simulation models for the assessment of the internal doses from diets have not been developed. Although regulation such as restricted food distributions is expected to effectively protect the public from radiation [6], such assessments have not been performed well.

The regulatory values for foods are generally determined from the intervention level of a dose, the age-specific daily consumption rates of foods, the age-specific committed dose coefficient, and the dilution factors (i.e., the ratios of contaminated foods to consumed foods) under the assumption that the concentrations of radionuclides in foods are constant or decayed [13]. In particular, the effectiveness of regulation and the establishment of a regulation value itself largely depend on the dilution factor (e.g., using a 1% dilution factor and 1 mSv as the intervention level yields the same regulation values as using a 100% dilution factor and 100 mSv as the intervention level). For the guideline levels of radionuclides in the first year after an accident by the Codex Alimentarius Commission, a dilution factor of 10% was applied on the basis of the ratio of the imported amounts to the production amounts [14]. The European Union and the US employs 10% and 30% dilution factors, respectively [13]. However, detailed studies on the estimation of the dilution factor have been limited. Furthermore, as the International Commission on Radiological Protection (ICRP) has adopted the fundamental concept of optimization of protection [15], it is also scientifically and socially required to evaluate the cost effectiveness of regulation. While stakeholder involvement and ethical consideration have recently become important, especially in post-accident recovery [13,16], a cost effectiveness analysis is essential as emergency preparedness.

This study had two objectives. First, we developed a model to estimate the intake of radionuclides from diets (excluding tap water) and to evaluate the effects of regulation (e.g., restrictions on the distribution of foods) in three different areas (Fukushima City, Tokyo, and Osaka; Figure S1) after the Fukushima accident and in virtual accident scenarios. Second, we evaluated the dilution factors and cost-effectiveness of regulation as basic information for setting regulatory values. This paper was prepared after an English translation and the addition of re-analyses, results, and discussion to abstracts presented at annual meeting/conferences [1,2,3].

## 2. Methods

### 2.1. Model Development

Radionuclide intake from foods can be explained in three steps: (1) transport of radionuclides from a power plant into the air and their deposition on the ground, (2) transfer of these radionuclides from the ground to foods, and (3) shipment of these foods from product areas to markets.

We simulated the deposition data by using the transport model for virtual accident scenarios. Details and the validity of the transport model were reported previously [12]. Two emission conditions were used: actual and maximum emission conditions. For the actual emission conditions, the deposition amounts were estimated from 11 March 2011 to 31 March 2011 by using the emission conditions that were considered to actually occur at the Fukushima accident (4.2 × 10^13^–8.9 × 10^15^ Bq h^−1^ for ^131^I; 4.2 × 10^11^–8.9 × 10^14^ Bq h^−1^ for ^137^Cs) [9] with the climate conditions in 2011. For the maximum emission conditions, the emission rate was set to the maximum value in the previous report (4.0 × 10^15^ Bq h^−1^ for ^131^I; 4.0 × 10^14^ Bq h^−1^ for ^137^Cs) [10], and the deposition amounts from 1 March to 31 March were estimated by using five sets of climate conditions in 2009–2013, as a sensitivity analysis [12]. The total bulk amounts (the sum of the dry and wet depositions) during the simulation periods were assumed to be deposited on a reference time of 0:00 16 March 2011 because the contamination of foods mostly started owing to wet deposition during the late night of 15 March [17]. Details of the models were described in supplementary materials (Method S1).

We estimated the ratios of the radionuclide concentrations in foods to their deposition in an individual municipality on the basis of the data observed from the Fukushima accident (Table S1). The median values were used to estimate the ratio of the concentration to deposition. The observed radionuclide concentrations in foods were determined according to >130,000 food- [18] and rice-monitoring data [19]. The radionuclide concentrations in foods were projected in consideration of the reduction coefficients described below from the observed data at the reference time of 0:00 16 March. Foods were divided into 29 categories in accordance with the previous study [6]. Tap water was excluded in this study. The reduction coefficients for radiocesium in each food were estimated from an exponential function of the concentration and elapsed time (*t* = 0 on 16 March 2011) for the same municipality (Figure S2). The data for “naganegi onion, chive, and asparagus” were not available; therefore, the median values of other leafy vegetables (i.e., “spinach”, “garland chrysanthemum, and ging-geng-cai”, “mustard spinach and nonheading lettuce”, “heading leafy vegetables”, and “broccoli and cauliflower”) were used. Physical decay coefficients [20] were used for all foods for radioiodine and some foods that did not show a positive value for the coefficient in the estimate above (Table S1). The data for the deposition of radioiodine (*n* = 597) were taken from our original measurements (*n* = 19), determined with the method previously reported [21], in addition to the data in previous reports [17,22,23]. The data for the deposition of radiocesium (*n* = 1833) were taken from the data for the Fukushima and Ibaraki prefectures in a previous report [24]. Since the ratio of the concentration to deposition of radiocesium for “garland chrysanthemum and ging-geng-cai” was not available, the value for “naganegi onion, chive, and asparagus” was used. Although we expected that the ratio of the concentration to deposition for each food can be explained by the transfer factor [25], the relationship was not significant (Figure S3), probably because phenomena other than transfer from soils to foods (e.g., direct deposition on foods) may govern the radionuclide concentrations in foods.

We then used the total bulk amount simulated in the transport model and the ratio of the concentration to deposition estimated above (Table S1) to calculate the radionuclide concentrations in each food collected for individual municipalities in 16 prefectures (Hokkaido, Aomori, Iwate, Miyagi, Akita, Yamagata, Fukushima, Ibaraki, Tochigi, Gunma, Saitama, Chiba, Kanagawa, Tokyo (Metropolis), Yamanashi, and Shizuoka; Figure S1). Since the depositions of ^134^Cs were similar to those of ^137^Cs [26,27], the ^134^Cs concentrations in foods were considered to be the same as those of ^137^Cs at 0:00 16 March. The radionuclide concentrations in foods since then were corrected with the reduction coefficients above (Table S1). We then estimated the dietary intake of radionuclides in the first year after the accident considering the regional trade of foods (i.e., arrival shares: fraction of a food in a targeted market (Fukushima City, Tokyo, or Osaka) from each municipality and overseas country [6]).

$${\mathrm{Dose}}_{k}(µ\mathrm{Sv})=A_{k}\times \sum_{i}\sum_{j}\sum_{t}(B_{i}\times C_{kijt}\times D_{ij}/100)$$

where *k* is the radionuclide, A*_k_* is the dose coefficient for radionuclide *k* (µSv Bq^−1^) [28,29]), *i* is the individual food category, *j* is the production area, *t* is the number of days after the accident (consumption date), *B_i_* is the daily consumption of food *i* per person (g d^−1^) [6], C_kijt_ is the concentration of radionuclide *k* in food *i* in area *j* at *t* days after the accident (Bq g^−1^), and D*_ij_* is the arrival share (the fraction of food *i* in the market that comes from area *j*) (%).

To estimate the ratio of food production among individual prefectural municipalities, we used the number of livestock [30], the cultivated areas of fields and rice paddies [31], the number of management bodies for mushrooms [32], and the number of grids (i.e., areas) in the municipalities for fisheries. The production of marine products in Fukushima Prefecture was regarded to be nil owing to the tsunami damage and the suspension of fishing, similar to the previous study [6].

This assessment can be regarded as conservative because (1) the total amounts until 31 March were regarded to be deposited at 0:00 16 March, (2) the decay of the concentration from collection to consumption (generally 1–2 days) was not considered, and (3) all contaminated foods were assumed to be consumed after 16 March.

### 2.2. Effects of Regulation

The effects of regulation were estimated from a comparison of the doses in the absence and presence of regulation. Regulation followed the actual applications after the Fukushima accident. The arrival shares of foods from five municipalities, in which most areas are located within 20 km of the Fukushima Daiichi Nuclear Power Station (i.e., Tomioka Town, Ookuma Town, Futaba Town, Namie Town, and Naraha Town; Figure S1), were assumed to be zero because the people in these areas were considered to be evacuated. Three cases were considered for the regulation of restricted food distributions. For Case 1, the distribution of foods collected in municipalities was restricted if the estimated median concentrations of radionuclides exceeded regulation values. The regulation values followed the provisional regulation values in Japan: ^131^I: milk and dairy products, 100 Bq kg^−1^ (the value for infants was applied); vegetables excluding root vegetables and tubers, 2000 Bq kg^−1^; fishery products, 2000 Bq kg^−1^; ^134^Cs + ^137^Cs: milk and dairy products, 200 Bq kg^−1^; vegetables, 500 Bq kg^−1^; grains, 500 Bq kg^−1^ (for rice, 100 Bq kg^−1^ was applied because of voluntary withholding); fishery products, 500 Bq kg^−1^; meat, egg, and other, 500 Bq kg^−1^. Regulation was assumed to start on 21 March. For radioiodine, regulation was considered to be lifted after 14 days since the concentrations of radionuclides in foods did not exceed regulation values. For radiocesium, regulation was considered to be lifted after 30 days since the concentrations of radionuclides in foods did not exceed regulation values (the ^137^Cs concentration did not exceed 1/2 of the regulation values). These lengths followed the actual practice at the Fukushima accident [33]. Foods in the municipalities, which were restricted either for radioiodine or radiocesium, were assumed to not be distributed to market.

At the Fukushima accident, the foods in the targeted municipalities were regulated if the concentrations of radionuclides in one of the measured foods exceeded the regulation values. Because the radionuclide concentrations in the same areas were 40–450% of the relative standard deviation (generally <200%) [6], we considered two other cases: the distribution of foods collected in municipalities was restricted if the estimated median concentrations of radionuclides exceeded 1/2 (Case 2) and 1/5 (Case 3) of the regulation values.

### 2.3. Dilution Factors

Dilution factors were estimated from the ratio of doses in Fukushima City considering the regional trade of foods to those assuming the ingestion of foods only in the most contaminated municipality. Fukushima City, the prefectural capital, was targeted because restricted food distribution was used for market regulation, and Fukushima City is the nearest large city to the nuclear power plant. For marine products in the case of ingestion of foods only in the most contaminated municipality, people were regarded to consume foods from Ibaraki Prefecture. To evaluate the dilution factors, the effects of restricted food distribution were not considered.

### 2.4. Cost and Effectiveness of Regulation

The cost and effectiveness of regulation on restricted food distribution were estimated for various regulatory values. The effectiveness of regulation was estimated from the difference in the doses with and without consideration of the regulation of restricted food distribution (Fukushima City, Case 1). Regulation of evacuation was considered for both. The regulatory values for radioiodine and radiocesium were set separately in the range of 1–20,000 Bq kg^−1^. The same value was set for all types of foods. The cost of restricted food distribution represented one of the foods produced but discarded (i.e., opportunity losses) and originated from previous reports (Table S2) [7,34,35,36]. Briefly, the cost was estimated from the unit cost of food in each category, the daily consumption of the food, the arrival share, and the number of days that food distribution was restricted in the targeted area.

The net values (i.e., benefit minus cost) for radiocesium were estimated by conversion from the effectiveness (mSv) to the benefit (Yen). The life years saved (LYS) from the reduced doses were considered as 0.0011 year mSv^−1^, which was the average value for 20-year-old men and women [37]. This value is similar to the estimate (of 0.0012 year mSv^−^^1^) ascribed to men and women aged 20–29-years in the Japanese population [38]. The value of a statistical life (350 million Yen), estimated from the Japanese WTP for the avoidance of risk [39], was converted to a value per LYS under the assumption that the LYS per mortality due to risk avoidance is 40 year [7]. The value was estimated to be 9700 Yen mSv^−1^. The net values for radioiodine were not applied because of a lack of WTP for the avoidance of risk of nonfatal cancer (e.g., thyroid cancer), which radioiodine specifically posed.

Since this analysis targeted markets in Fukushima City, the nearest large city to the nuclear power plant, the net values for the nationwide average should be lower than those estimated. However, we used these values because we considered that the values in the affected areas rather than the nationwide average should be chosen to determine the regulatory values.

## 3. Results

### 3.1. Validation of Developed Model

To validate the developed model, we compared the estimated effective doses of radiocesium under actual emission conditions with the observed doses through food-duplicate or market-basket surveys [18,38,39] (Table 1). As described in Methods, validation of the regional trade of foods and the transport model was reported elsewhere [6,12]. A unit of μSv month^−1^ was used since the dose might be subject to temporary change. While the effective doses slightly differed among the three cases and were reduced at times, especially in Fukushima City, the observed data also showed variations (i.e., relative standard deviation: 77–195%). The estimated doses generally agreed within a factor of three with those observed when these variations were taken into consideration. Since the observed data in food-duplicate or market-basket surveys were not available just after the accident, we compared the effective doses of radioiodine and radiocesium estimated in this study in the first year after the accident with those previously reported [6], which were estimated from the observed concentrations of food categories in each municipality and the regional trade of foods (Figure S4). The doses estimated in this study were comparable to or slightly higher than those in the previous study.

### 3.2. Effective Doses and Effects of Regulation

**Figure 1:**
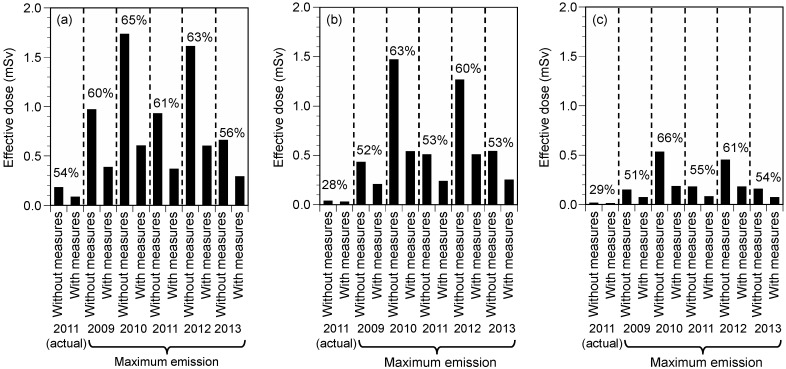
Effective doses for men (≥19 year) in the first year and the effectiveness of measures under different climate conditions without and with measures (Case 1): (**a**) Fukushima City, (**b**) Tokyo, and (**c**) Osaka. Percentages represent reduction ratios. Measures include restrictions on food distribution and evacuation.

The effective doses of the sum of radioiodine and radiocesium in the first year were simulated with and without measures (Figure 1). The effective doses in Fukushima City with and without measures under actual emission conditions were 0.08 and 0.18 mSv, respectively, exhibiting a reduction ratio of 54%. Similarly, the effective doses in Fukushima City without measures under maximum emission conditions ranged from 0.66 mSv to 1.73 mSv, whereas those with measures were in the range of 0.29–0.60 mSv. Measures resulted in reductions in doses of 56–65% under maximum emission conditions. The effective doses in Tokyo and Osaka were lower than those in Fukushima City; however, the reduction ratios were almost comparable among three areas, except under actual emission conditions (28% in Tokyo and 29% in Osaka).

### 3.3. Dilution Factors and Cost and Effectiveness of Regulation

**Figure 2:**
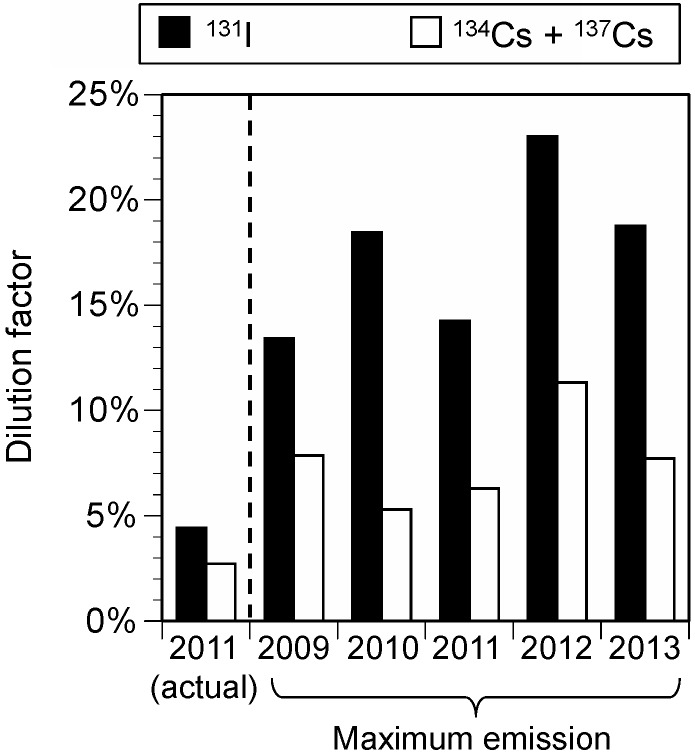
Dilution factors of radioiodine and radiocesium (men, ≥19 year).

The estimated dilution factors under actual emission conditions were 4.4% for radioiodine and 2.7% for radiocesium (Figure 2). The dilution factors under maximum emission conditions were 13–23% for radioiodine and 5.3–11% for radiocesium.

**Figure 3:**
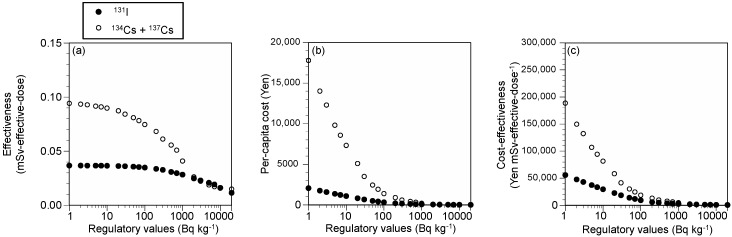
Cost and effectiveness of restricted food distribution for each regulatory value: (**a**) reduction in effective doses, (**b**) per-capita cost, and (**c**) cost effectiveness. Men, ≥19 year, actual emission conditions, Case 1.

The cost and effectiveness of restricted food distribution were then estimated depending on the regulatory values (Figure 3). The effectiveness increased with strict regulatory values; however, the increase in the effectiveness gradually weakened for both radioiodine and radiocesium. On the contrary, the cost of restricted food distribution sharply increased with strict regulatory values, especially for radiocesium. Consequently, strict regulation worsened the cost-effectiveness for both radionuclides.

**Figure 4:**
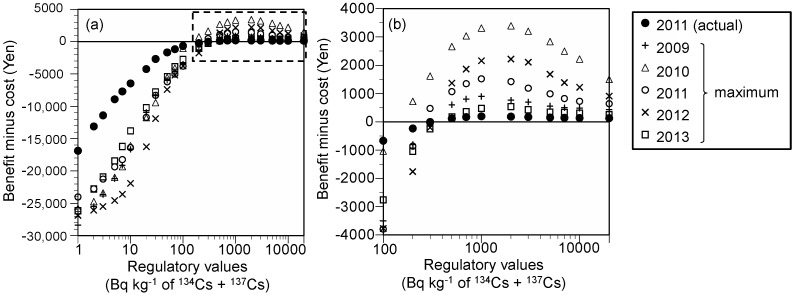
Net values of restricted food distribution for each regulatory value for radiocesium: (**a**) 1–20,000 Bq kg^−1^ and (**b**) 100–20,000 Bq kg^−1^.

The net values were then estimated by converting the effectiveness to the benefit by using the willingness to pay (WTP) for the avoidance of risk (Figure 4). The net values for radiocesium showed negative values at <200–500 Bq kg^−1^, irrespective of all conditions.

## 4. Discussion

This study developed a model for estimating the dietary intake of radionuclides considering the regional trade of foods for the purposes of emergency preparedness and the establishment of regulations on food distribution. The estimated doses were in agreement with the observed data, demonstrating the reliability of the model (Table 1 and Table S4). Although a decreasing trend of the doses was suggested, it was not possible to account for temporal changes in the doses based on the data in which variations were reflected. Thus, further studies are warranted to elucidate seasonal or temporary dose-related changes.

By using the developed model, we evaluated the variations in the doses from the intakes of radionuclides and the effectiveness of regulation. Under the maximum emission conditions for five climates, the ratios of the maximum to minimum values without measures were 2.63 for Fukushima City, 3.41 for Tokyo, and 3.61 for Osaka, whereas those with measures were 2.08, 2.63, and 2.57, respectively (Figure 1). This suggests that the climate conditions are important factors governing the radiation exposure doses from the intakes of diets, and measures reduce the variations in doses due to climate conditions.

The dilution factors were estimated to be 4.4% for radioiodine and 2.7% for radiocesium under actual conditions (Figure 2). It is noted that the Japanese provisional regulation values were set at 100% (no dilution) for radioiodine and 50% for radiocesium [13]. These results indicate that the Japanese provisional regulation values were conservatively set by a factor of approximately 20 with respect to the dilution factors. Under maximum emission conditions, the dilution factors were 13–23% for radioiodine and 5.3–11% for radiocesium; these values were larger than those under actual emission conditions. This is because a constant rate of emission was assumed under maximum emission conditions, resulting in smaller spatial variations in deposition. Even under such extreme conditions, the dilution factors were smaller than those used in the Japanese provisional regulation values. This finding indicates the importance and need to carefully set the dilution factors for the establishment of regulatory values.

The developed model is also beneficial for estimating the cost and effectiveness of regulation. This study presented an example of a cost-effectiveness analysis against regulatory values; stricter regulation resulted in a worse cost-effectiveness (Figure 3). This result highlights the importance of the concepts of optimization of protection and as low as reasonably achievable [15]. These concepts do not intend to maximize the net values; rather, negative values of net values were not justified from the viewpoint of a cost–benefit balance. The net values for regulatory values of 200–500 Bq kg^−1^ for ^134^Cs + ^137^Cs were higher than 0 and were regarded as appropriate. The cost and the public’s WTP for the avoidance of risk do not justify stricter regulatory values than these values. The regulatory values of 200–500 Bq kg^−1^ for ^134^Cs + ^137^Cs were comparable to the provisional regulation values applied after the Fukushima accident (i.e., milk and dairy products: 200 Bq kg^−1^; vegetables, grains, fishery products, meat, egg, and other: 500 Bq kg^−1^). Likewise, the cost–benefit approach based on the developed model has utility for setting the regulatory values.

This study had some limitations. First, since we aimed to develop the model based on the Fukushima Nuclear Power Station accident, we have limited ability to generalize the findings in other regions. Second, seasonal variations were not considered. Third, we excluded the intake of radionuclides from tap water. Future studies are necessary to develop an integrated model incorporating intakes from tap water with consideration of seasonal and spatial variations.

## 5. Conclusions

In summary, we developed a model for estimated doses from the intakes of radionuclides with the consideration of the regional trade of foods. This model enabled us to assess the doses from the intake of diets for residents living in different areas and the effectiveness of regulation on the basis of input data regarding the amounts of radionuclides emitted from a nuclear power plant and climate conditions. The developed model is useful for providing basic information regarding the setting of regulatory values such as dilution factors and the cost-effectiveness of regulation. This study highlighted the significance and utility of the model in a risk analysis for emergency preparedness and regulation due to the global increase in its importance.

## Figures and Tables

**Table 1:** Estimated and observed effective doses for men (≥19 y) [μSv month^−1^]: (a) Fukushima City (or Fukushima Pref.), (b) Tokyo (or Kanto), and (c) Osaka. Observed values are taken from food-duplicate and market-basket surveys [18,40,41]. Case 1, the distribution of foods was restricted if the estimated median concentrations of radionuclides exceed regulation values. Case 2, median > 1/2 × regulation values. Case 3, median > 1/5 × regulation values.

	Jul. 2011	Sep.–Nov. 2011	Dec. 2011	Feb.–Mar. 2012
Fukushima City Case 1 (this study)	3.38	1.54	1.26	1.12
Fukushima City Case 2 (this study)	2.40	1.40	1.13	1.02
Fukushima City Case 3 (this study)	1.36	1.07	0.84	0.73
Fukushima Pref. (ref [41]) ^a^ (*n* = 55)	0.53 ± 1.04 ^e^ (not detected–6.93) ^f^	-	-	-
Fukushima Pref. (ref [18]) ^b^	-	1.58	-	-
Fukushima Pref. (ref [40]) ^a^ (*n* = 26)	-	-	2.17 ± 1.67 ^e^ (<0.22–8.25) ^f^	-
Nakadori, Fukushima Pref. (ref [18]) ^b^	-	-	-	0.55
Tokyo Case 1 (this study)	-	0.70	0.63	0.59
Tokyo Case 2 (this study)	-	0.69	0.62	0.57
Tokyo Case 3 (this study)	-	0.60	0.54	0.50
Tokyo (ref [18]) ^b^	-	0.18	-	-
Kanto (ref [40]) ^a,c^ (*n* = 16)	-	-	0.92 ± 1.42 ^e^ (<0.11–5.00) ^f^	-
Kanto (ref [18]) ^b,d^	-	-	-	0.43
Osaka Case 1 (this study)	-	-	-	0.34
Osaka Case 2 (this study)	-	-	-	0.33
Osaka Case 3 (this study)	-	-	-	0.29
Osaka (ref [18]) ^b^	-	-	-	0.13

^a^ food-duplicate survey. ^b^ market basket survey. ^c^ including Tochigi, Gunma, Ibaraki, Saitama, Chiba, Tokyo, Kanagawa, and Nagano prefectures. ^d^ including Tochigi, Ibaraki, Saitama, and Kanagawa prefectures. ^e^ arithmetic mean ± standard deviation. ^f^ minimum–maximum.

## Supplementary Materials

The following are available online at http://www.mdpi.com/1660-4601/15/8/1589/s1, Method S1: Transport model details, Figure S1: Locations, Figure S2: Estimates of the reduction coefficient and the ratio of the concentration to deposition (radiocesium in spinach), Figure S3: Relationship between the transfer factor and the ratio of the concentration to deposition of radiocesium estimated in this study, Figure S4: Comparison of the results obtained in this study with those in another study, Table S1: Reduction coefficients and ratios of the concentration to deposition, Table S2: Unit costs for restricted food distributions.
