# Supplementary material for: Estimation of Dietary Intake of Radionuclides and Effectiveness of Regulation after the Fukushima Accident and in Virtual Nuclear Power Plant Accident Scenarios
Source: Int J Environ Res Public Health. 2018 Jul 26;15(8):1589. doi: 10.3390/ijerph15081589 (PMC6121232; doi:10.3390/ijerph15081589)
Supplement: Supplementary file 1 [file ijerph-15-01589-s001.pdf]

Method S1. Transport model details

We conducted hindcast simulations from 11 to 31 March 2011 [1] using the Isotopic Regional Spectral Model [2, 3], and the time-varying release rates were estimated using the reverse-estimation method [4]. The height of the emission source was set to the surface, because the emission height was estimated to range from 20 to 120 m (fairly close to the surface) in the reverse-estimation method [4-6]. The mesoscale model grid point value datasets, which were provided by the Japan Meteorological Agency, served as the initial and lateral boundary conditions of the model. The spectral nudging method was applied to the lateral boundary data. The grid spacing was 5 km, and the number of vertical layers of the sigma coordinate system was 28. The horizontal and meridional ranges of the domain were 800 km and 950 km, respectively. A semi-Lagrangian model was used to calculate the transportation of radioactive materials [3].

The wet deposition (washout process) was calculated as:

$$dC/dt = -\alpha P/q C \qquad (1)$$

where C is the atmospheric concentration of radioactive materials; α is the washout coefficient (0.5); and P and q are the water condensation and the water vapour at each atmospheric layer, respectively [7].

The dry deposition was calculated as:

$$F_{dry} = V_d C_{(z=1)} \qquad (2)$$

where V_d is the deposition speed, and $C_{(z=1)}$ is the concentration in the lowest layer. The V_d values of ^{137}Cs and ^{131}I are 1×10^{-3} ms^{-1} and 5×10^{-3} ms^{-1} over the ocean and 5×10^{-3} ms^{-1} and 2.5×10^{-2} ms^{-1} over the land areas, respectively [8].

We also conducted long-term simulations with a fixed release rate in March from 2009 to 2013. The other calculation conditions were the same as in the hindcast simulation.

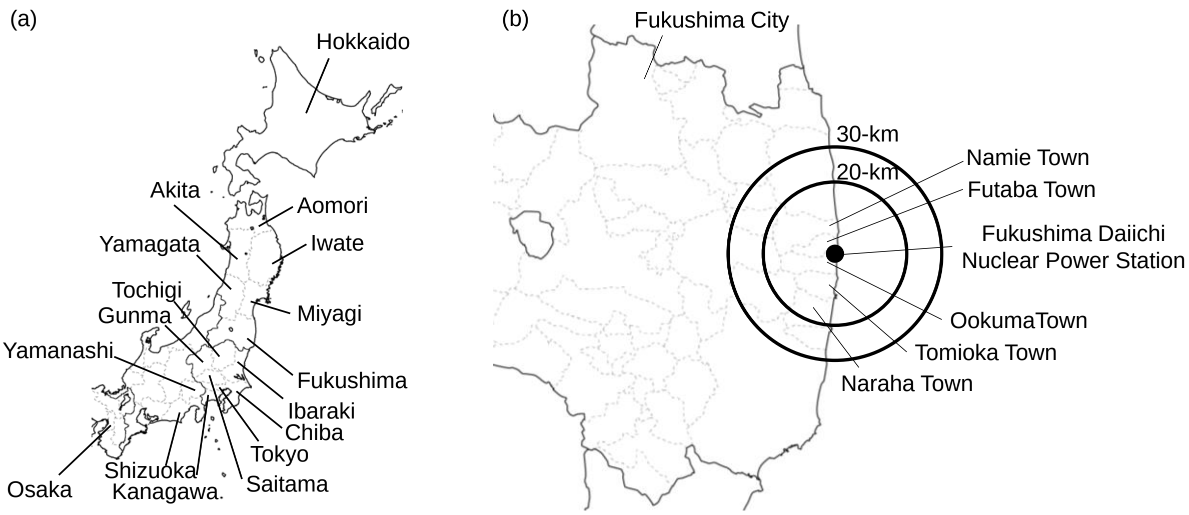

Figure S1. Locations: (a) prefectures in Japan and (b) municipalities in Fukushima Prefecture. Figure was prepared by using source map files obtained from *Hakuchizu-senmonten*: http://www.freemap.jp/.

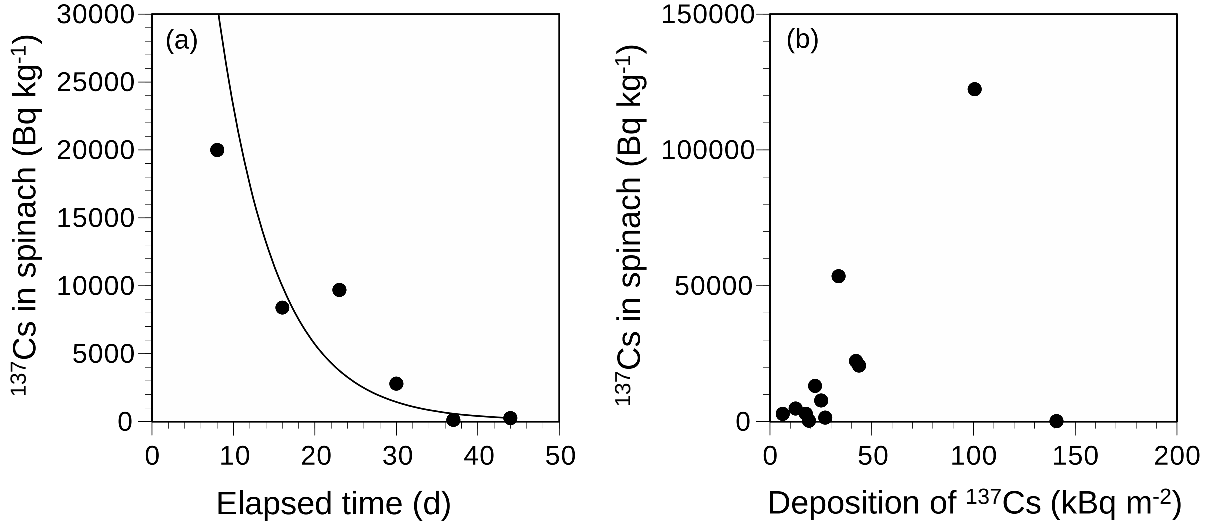

Figure S2. Estimates of the reduction coefficient and the ratio of the concentration to deposition (radiocesium in spinach). (a) ^{137}Cs concentration in spinach versus the elapsed time. r^2 = 0.83. The reduction coefficient is 0.141 d^{-1}. (b) ^{137}Cs concentration in spinach versus the deposition of ^{137}Cs at 0:00 March 16, 2011.

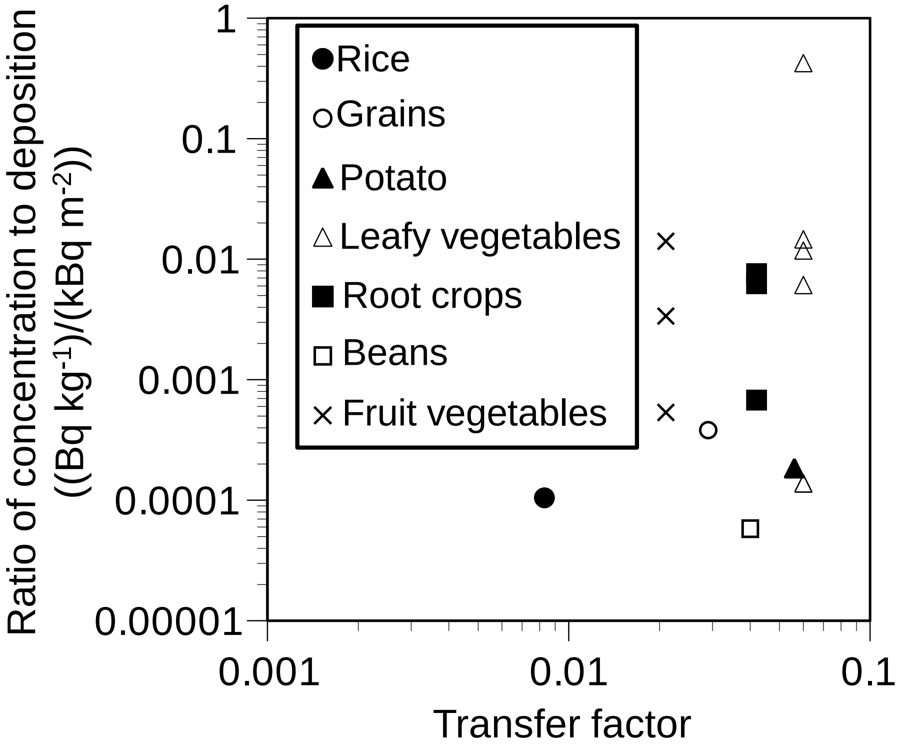

Figure S3. Relationship between the transfer factor and the ratio of the concentration to deposition of radiocesium estimated in this study.

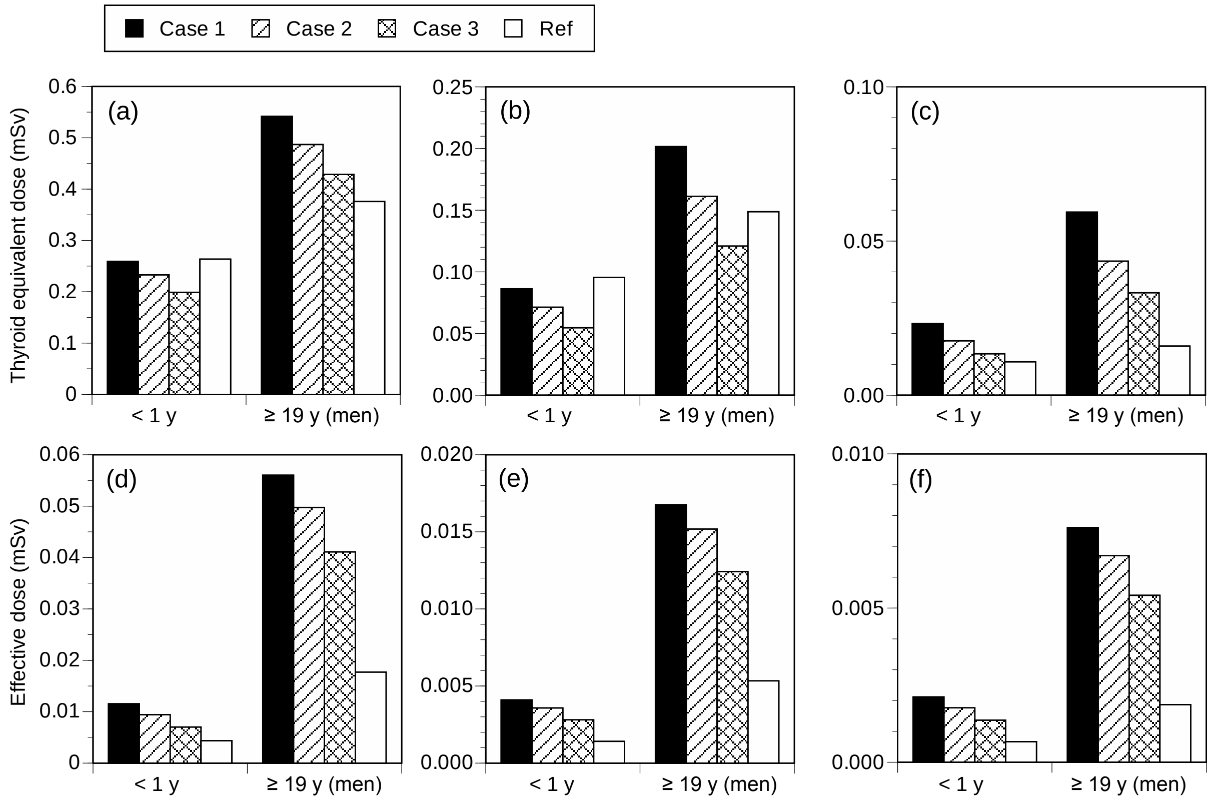

Figure S4. Comparison of the results obtained in this study with those in another study [9]: (a) radioiodine, Fukushima City; (b) radioiodine, Tokyo; (c) radioiodine, Osaka; (d) radiocesium, Fukushima City; (e) radiocesium, Tokyo; and (f) radiocesium, Osaka.

Table S1. Reduction coefficients and ratios of the concentration to deposition.

	Reduction coefficient (d^{-1})			Ratio of concentration to deposition (($Bq\ kg^{-1}$)/($kBq\ m^{-2}$))	
	^{131}I	^{134}Cs	^{137}Cs	^{131}I	^{134}Cs or ^{137}Cs
Rice	0.0864	0.000919	6.29×10^{-5}	-	0.000105
Grains	0.0864	0.000919	6.29×10^{-5}	-	0.000382
Potato	0.0864	0.000919	6.29×10^{-5}	-	0.000185
Spinach	0.0864	0.140	0.141	0.0296	0.424
Garland chrysanthemum and ging-geng-cai	0.0864	0.005	0.005	0.000495	0.000138
Mustard spinach and nonheading lettuce	0.0864	0.144	0.139	0.00447	0.00611
Heading leafy vegetables	0.0864	0.084	0.076	0.00329	0.0118
Broccoli and cauliflower	0.0864	0.049	0.046	0.001	0.0146
Naganegi onion, chivee, and asparagus	0.0864	0.084	0.076	0.000259	0.000138
Turnip	0.0864	0.073	0.067	0.00167	0.00625
Bamboo shoots	0.0864	0.019	0.016	-	0.0076
Other root crops	0.0864	0.000919	6.29×10^{-5}	-	0.000678
Beans	0.0864	0.004	0.004	-	5.82×10^{-5}
Kiwifruit	0.0864	0.000919	6.29×10^{-5}	-	0.000536
Chestnut	0.0864	0.013	0.014	-	0.0141
Other fruit vegetables	0.0864	0.023	0.024	5.79×10^{-5}	0.00338
Milk	0.0864	0.116	0.044	0.000155	0.000109
Dairy products	0.0864	0.116	0.044	0.000155	0.000109
Formula milk	0.0864	0.116	0.044	-	-
Beef	0.0864	0.000919	6.29×10^{-5}	-	0.000166
Pork	0.0864	0.008	0.008	-	6.92×10^{-5}
Chicken	0.0864	0.000919	6.29×10^{-5}	8.3×10^{-6}	0.000308
Chicken eggs	0.0864	0.000919	6.29×10^{-5}	0.000262	-
Wild *ayu*, wild Japanese dace and wild landlocked *masu* salmon	0.0864	0.008	0.007	-	0.00826
Other fresh fisheries products	0.0864	0.006	0.005	6.47×10^{-5}	0.00611
Marine products	0.0864	0.000919	6.29×10^{-5}	-	0.000858
Teas	0.0864	0.020	0.019	-	0.106
Shiitake mushroom	0.0864	0.000919	6.29×10^{-5}	-	0.00125
Other mushrooms	0.0864	0.000919	6.29×10^{-5}	3.56×10^{-5}	0.000184

Table S2. Unit costs for restricted food distributions.

	Unit cost (Yen/kg)
Rice	230
Grains	88
Potato	77
Spinach	260
Garland chrysanthemum and ging-geng-cai	310
Mustard spinach and nonheading lettuce	130
Heading leafy vegetables	31
Broccoli and cauliflower	170
Naganegi onion, chivee, and asparagus	250
Turnip	72
Bamboo shoots	47
Other root crops	43
Beans	290
Kiwifruit	180
Chestnut	120
Other fruit vegetables	160
Milk	54
Dairy products	54
Formula milk	54
Beef	470
Pork	190
Chicken	120
Chicken eggs	170
Wild *ayu*, wild Japanese dace and wild landlocked *masu* salmon	1410
Other fresh fisheries products	900
Marine products	430
Teas	140
Shiitake mushroom	580
Other mushrooms	270

References

1. Yoshikane, T.; Yoshimura, K.; Chang, E. C.; Saya, A.; Oki, T., Long-distance transport of radioactive plume by nocturnal local winds. *Sci. Rep.* **2016,** *6*, 36584.
2. Yoshimura Kei; Kanamitsu Masao; Dettinger Michael, Regional downscaling for stable water isotopes: A case study of an atmospheric river event. *J Geophys Res* **2010,** *115*.
3. Chang, E.-C.; Yoshimura K., A semi-Lagrangian advection scheme for radioactive tracers in the NCEP Regional Spectral Model (RSM). *Geosci. Model Dev.* **2015,** *8*, 3247-3255.
4. Kobayashi, T.; Nagai, H.; Chino, M.; Kawamura, H., Source term estimation of atmospheric release due to the Fukushima Dai-ichi Nuclear Power Plant accident by atmospheric and oceanic dispersion simulations. *J. Nucl. Sci. Technol.* **2013,** *50*, 255-264.
5. Chino, M.; Nakayama, H.; Nagai, H.; Terada, H.; Katata, G.; Yamazawa, H., Preliminary estimation of release amounts of ^{131}I and ^{137}Cs accidentally discharged from the Fukushima Daiichi Nuclear Power Plant into the atmosphere. *J. Nucl. Sci. Technol.* **2011,** *48*, 1129-1134.
6. Terada, H.; Katata, G.; Chino, M.; Nagai, H., Atmospheric discharge and dispersion of radionuclides during the Fukushima Dai-ichi Nuclear Power Plant accident. Part II: verification of the source term and analysis of regional-scale atmospheric dispersion. *J. Environ. Radioactiv.* **2012,** *112*, 141-154.
7. Saya, A.; Yoshimura, K.; Oki, T., Simulation of radioactive tracer transport using isorsm and uncertainty analyses. *J JSCE* **2015,** *3*, 60-66.
8. Maryon, R. H.; Smith, F. B.; Conway, B. J.; Goddard, D. M., The U.K. nuclear accident model. *Prog Nucl Energy* **1991,** *26*, 85-104.
9. Murakami, M.; Oki, T., Estimated dietary intake of radionuclides and health risks for the citizens of Fukushima City, Tokyo, and Osaka after the 2011 nuclear accident. *Plos One* **2014,** *9*, e112791.
